# The Functions of the Mammalian Methionine Sulfoxide Reductase System and Related Diseases

**DOI:** 10.3390/antiox7090122

**Published:** 2018-09-18

**Authors:** Beichen Jiang, Jackob Moskovitz

**Affiliations:** Department of Pharmacology and Toxicology, School of Pharmacy, University of Kansas, Lawrence, KS 66045, USA; jiangbc@ku.edu

**Keywords:** methionine sulfoxide reductase, methionine oxidation, oxidative stress

## Abstract

This review article describes and discusses the current knowledge on the general role of the methionine sulfoxide reductase (MSR) system and the particular role of MSR type A (MSRA) in mammals. A powerful tool to investigate the contribution of MSRA to molecular processes within a mammalian system/organism is the *MSRA* knockout. The deficiency of MSRA in this mouse model provides hints and evidence for this enzyme function in health and disease. Accordingly, the potential involvement of MSRA in the processes leading to neurodegenerative diseases, neurological disorders, cystic fibrosis, cancer, and hearing loss will be deliberated and evaluated.

## 1. Introduction

**Figure 1:**
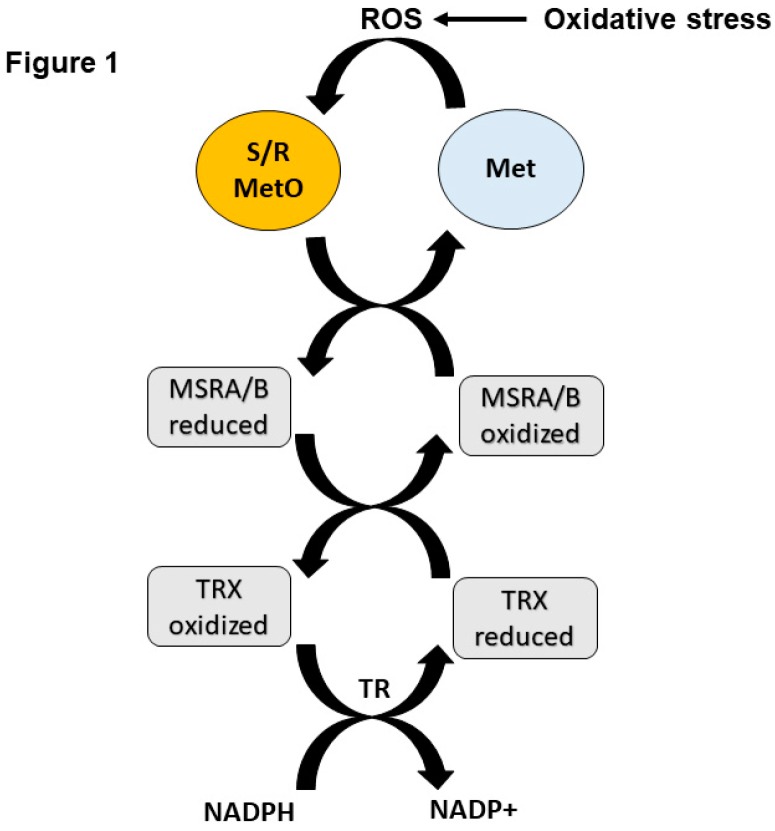
Schematic illustrating of the redox cycle of MSRA and MSRB in reducing MetO. Oxidative stress causes the production of reactive oxygen species (ROS) that in-turn oxidize Met to *S*-MetO and *R*-MetO epimers. The reduced form of either MSRA or MSRB reduces *S*- or *R*-MetO respectively. The oxidized forms of the MSR enzymes are reduced by the reduced form of thioredoxin (TRX). The oxidized form of TRX is reduced by the enzymatic activity of thioredoxin reductase (TR) that uses NADPH for its function, resulting in the formation of NADP^+^.

Environmental and cellular driven oxidative stress is manifested by the production of reactive oxygen species (ROS) that can be toxic to the cell/organism, which may lead to pathological consequences and death. To protect the cell from these toxic radicals, several antioxidant systems were developed within the cell and among them was the methionine sulfoxide reductase system [1]. This system is much conserved in evolution and comprises two types of MSRs: MSRA and MSRB that reduce oxidized methionine (methionine sulfoxide, MetO) in the form of *S*-MetO or *R*-MetO to methionine, respectively [1]. It is important to note that oxidative damage results in the formation of equal amounts of both MetO epimers. The suggestion that the MSR system can use various cellular reduction powers to reduce MetO is supported by in vitro activity assays. For example, reduced forms of either thioredoxin, glutathione, or DTT (Dithiothreitol) can serve as a reduction power for the MSR activity [1]. However, in most biological systems, thioredoxin serves as the natural reducing agent for MSR activity (Figure 1). Generally, the MSR system can affect any process that involves oxidative damage. These MSR enzymes possess the ability to reduce both MetO of both proteins and free amino acids, while there are other types of MSRs that reduce free-MetO only [1,2,3]. In eukaryotes, the MSRA can be translocated into the nucleus and mitochondria through alternative mRNA splicing and/or myristoylation processes, whereas MSRB1 is a selenoprotein that can be detected in the nucleus and cytoplasm [1,4,5]. The other two MSRBs, MSRB2 and MSRB3, are translocated into the mitochondria and MSRB3 can also be found in the endoplasmic reticulum [1,4]. The expression pattern of MSRA and MSRB1 are similar in mammalian organs, in which the highest expression levels are commonly detected in the liver and the kidney followed by the brain [6]. MSRA or MSRB can negatively regulate each other’s expression level when the expression of either one is reduced (i.e., *MSRA* knockout (*MSRA* KO) mouse exhibits lower levels of MSRB1 and *MSRB1* knockout mouse exhibits lower levels of MSRA) [7,8]. The *MSRA* KO mouse is more vulnerable to oxidative stress and demonstrates several molecular phenotypes that can be associated with age-associated diseases, depending on the tested organ, such as Alzheimer’s disease (AD) [9,10,11], and Parkinson’s disease (PD) [9,12]. Other malfunctions that are a consequence of MSRA’s absence in the *MSRA* KO mice may be related to mental health disorders [13,14], heart disease [15], liver toxicity [16], cancer [17,18], and cystic fibrosis (CF) (when the *MSRA* is ablated in the mouse model of CF) [19]. Although MSRA can influence various different cellular processes, so far, no particular pathway has been identified to be most affected by a malfunctioning MSRA under basal conditions, which will result in a clear disease development/progression. However, environmental, epigenetics, and aging may exacerbate the development of a specific disease once the function or expression of either MSRA or MSRB or both is compromised. In this review, the involvement of MSRA in protein regulation with respect to disease and aging will be discussed as well as the potential MSRA-based therapies against oxidative-stress related diseases.

## 2. MSRA and Protection Against Oxidative Stress in Mammals

The MSR system can scavenge ROS through the cycled reduction of MetO to methionine (Met). At the advanced age, the mammalian MSR activity is declined [20], and the lack of MSRA causes eukaryotic cells/organisms to be more sensitive to oxidative stress as measured by their reduced survival rate under this stressful environment [10,21,22]. Confirmation of the vulnerability of our *MSRA* KO mice to oxidative stress was confirmed by others while failing to reproduce the difference in longevity under normoxic conditions [23]. One crucial difference between the experimental procedures presented in these two studies is the breeding and selection of mouse strains participating in the analyses. In our survival experiment, we used the three strains (WT, *MSRA* KO and heterozygotes for the *MSRA* gene) that were originated from the same heterozygote parent strains. However, in the other study [23], each strain was obtained from an independent inbred breeding of each strain. The latter breeding strategy may strongly affect the survival curve due to not having the same parental breeders. In contrast, overexpression of MSRA has been shown to alleviate oxidative stress insult to human T-cell [24], fruit-fly [25], and worm [26], and protect neuronal cells from mitochondrial driven toxicity and neurotoxins [27]. It is yet to be determined and identified the nature of the compounds and molecules that specifically upregulate the expression of MSRA as a basis for the development of MSRA-dependent therapy. Currently, there are several examples of compounds that can upregulate the expression of MSRA and share a methyl-sulfoxide group in their structure [28], including free MetO [27]. This phenomenon suggests that the accumulation of MSRA substrates (containing methyl-sulfoxide moiety) participate in signal transduction pathways that control the regulation of MSRA expression.

In the next sections, the potential role of the MSR system and particularly MSRA will be discussed in the context of a specific disease.

## 3. MSRA and Neurodegenerative Diseases

### 3.1. MetO and MSRA and Alzheimer’s Disease (AD)

In the AD, levels of oxidative damage markers, including lipid peroxidation and nitration, nucleic acid oxidation, and protein carbonylation, are increased in vulnerable brain areas relative to age-matched healthy individuals [29]. Extracellular amyloid plaques comprising predominantly fibrillar amyloid *β*-protein (A*β*) characterize AD pathologically and intracellular neurofibrillary tangles made of hyperphosphorylated tau [30]. Oxidative stress is one of the earliest consequences of toxic insults mediated by soluble A*β* oligomers [31]. Mitochondria are particularly sensitive to oxidative stress, and reduced metabolic activity resulting from oxidative damage to vital mitochondrial components has been demonstrated in AD [32]. A single Met residue in A*β*, Met35, is located in the middle of the hydrophobic C-terminal region (A*β*, 29−42). Therefore, the dramatic increase in the polarity of the Met side chain that occurs upon oxidation has a profound effect on the hydropathic characterization of the entire region [33]. Met is highly susceptible to oxidation in-vivo, particularly under conditions of oxidative stress. The sulfoxide form has been found to comprise 10–50% of A*β* in amyloid plaques of AD brain [34,35,36,37]. Several laboratories have reported lower toxicity of A*β*-MetO relative to WT A*β* [38,39]. Our and other data suggest that the apparent lower toxicity of A*β*-MetO might result not only from an altered structure in the C-terminal region of A*β* or alteration of A*β* oligomerization [18,27], but also due to MSRA activation by A*β*-MetO, suggesting that the cells sense the presence of MetO in A*β* and upregulate MSRA to provide stronger cellular protection [33]. However, as the oxidative stress with advanced age is increased while the activity of the MSRA and other antioxidants are decreased [20], A*β*-MetO is predicted to gain toxicity towards neurons. Another important contributing factor to the enhanced toxicity of A*β*-MetO with age is its higher solubility in aqueous solution, providing better mobility and access of the A*β*-MetO to neurons throughout the hippocampal and cortical regions of the brain. Further supportive evidence for the protective role of MSRA against the appearance of AD markers are the increased levels of A*β*, phosphorylated tau, and damaged astrocytes in the hippocampal region of the *MSRA* KO mice [10]. Accordingly, clearance of A*β*-MetO from brain and/or reduction of MetO-proteins in the brain by either MSRA/B or both may prevent or reduce AD-associated cognitive decline.

### 3.2. MetO and MSRA and Parkinson’s Disease (PD)

One of the hallmarks of PD is the formation of neuronal inclusion bodies (Lewy bodies) that contain aggregated proteins in which α-synuclein fibrils are mostly present. The function of the α-synuclein protein and its involvement in the development and pathogenesis of PD is not fully understood. Methionine oxidation of the α-synuclein protein can alter its fibrillation and enhance its resistance to degradation, especially in the absence of MSRA [40]. This observation suggests that enhanced Met oxidation of α-synuclein and lack of MSRA contribute to the toxicity of posttranslational modified α-synuclein [40], which is associated with PD disease. Dopaminergic cells in culture have been shown to be protected from PD-related insults by the expression and function of MSRA [41,42]. In addition, gait disturbances including shortened stride length and abnormal stride frequency are symptomatic of PD. The *MSRA* KO mice exhibit a reduced stride length and stride duration as well as an increased stride frequency compared with control mice [14]. These decreased parameters suggest a lack of motor control that evidently has a similar pattern to gait performance demonstrated by MPTP (1-methyl-4-phenyl-1,2,3,6-tetrahydropyridine)-injected mice [43] a common PD mouse model. These observed gait disturbances of the MSRA KO mice are exacerbated with age that plays a major role in the development of sporadic PD [11]. In conclusion, it is proposed that the upregulation of MSRA before and during the manifestation of PD may alleviate symptoms that are associated with this disease. More research is required to discover the compounds that can cause an increase in MSRA activity, especially in dopaminergic neurons, and be used as a possible therapy for PD patients.

### 3.3. MetO and MSRA and Dopamine-Related Neurological Abnormalities

The *MSRA* KO mice exhibit high striatal dopamine (DA) levels at a young mature age, without showing higher locomotor activity in comparison to wild type (WT) cohorts [12]. Further investigations of this observation led to the discovery that one of the causes that may influence this discrepancy is the compromised function of D2-DA receptor (D2DR) of the *MSRA* KO mice [44]. These data suggest that the *MSRA* KO mice are exposed to enhanced oxidative stress (due to the lack of MSRA), resulting in enhanced Met oxidation of proteins including D2DR. Accordingly, this situation may prompt enhance synthesis of DA to compensate for the inefficient function of DA-related proteins, such as D2DR. High levels of striatal DA is known to be linked to mood disorders [14] but it has to be evaluated case by case as the ratio between the prefrontal cortex and striatal DA plays an important role in the mental state diagnostics. Brain DA levels are elevated through either or reduced degradation or both. The *MSRA* KO possess elevated levels and activity of 14-3-3 zeta that activated tyrosine hydroxylase, leading to increased production of DA [12]. Complementarily, the activity of the catechol-*O*-methyl transferase (COMT) (one of the enzymes that degrade DA) is reduced in the *MSRA* KO mice [13]. Taken together, it is suggested that MSRA plays an important role in the regulation of brain dopamine. A supportive evidence for the interaction between MSRA and COMT in mice was found in our studies using postmortem human sections containing a known polymorphism of COMT Val (108/156) Met [14]. In these studies, we have revealed that homozygous carriers of Met, but not Val alleles, the activity of COMT and MSR are significantly correlated throughout all tested brain regions. These data suggest that the reduced enzymatic activity of Met-containing COMT may be secondary to Met oxidation and point to MSR as a key molecular determinant for the modulation of COMT activity in humans. Thus, identifying compounds that can either upregulate the expression or enhance the activity of MSRA may be used to increase COMT activity as a therapeutic intervention. Evidently, more research is required to establish the impact of MSRA activity on COMT with respect to neurological diseases/disorders.

### 3.4. MSRA and Cystic Fibrosis (CF)

Cystic fibrosis is an autosomal recessive condition caused by mutations in the cystic fibrosis transmembrane conductance regulator (CFTR; MIM 602421) gene. The earliest symptom of CF is the digestion problem in the small intestine (meconium ileus (MI), a prenatal obstruction of the small intestine at the ileocecal junction). To identify MI modifier genes in human, CF mouse models have been utilized. Mice with disruption of *Cftr* present with an MI-like phenotype, which differs from human MI in several respects and mainly the fact that CF mice die shortly after birth [19]. Studies in humans suggest that intestinal obstruction due to the loss of CFTR function is a consistent observation. However, that is not presented in all CF patients and suggests that other genetic factors may play a role in the manifestation of the disease. A regional family-based association analysis of a locus that is linked to MI has identified SNP haplotypes within the *MSRA* gene [19]. Cross-breeding between *MSRA* KO and CF mice showed that intestinal obstruction at the time of weaning was decreased in CF mice with *MSRA* null alleles compared with WT cohorts, resulting in significant improvement in survival [19]. Accordingly, inhibition of MSRA expression and/or activity may provide a new treatment for CF, following further knowledge gathering about the interaction between the MSR system and the CFTR function.

### 3.5. MSRA and Liver and Kidney Toxicity

MSRA is highly expressed in liver and kidney and the hypothesis is that this feature enables these organs to efficiently detoxify methyl-MetO-containing molecules from the body. Recent studies have suggested that MSRA and MSRB1 protect hepatocytes from the toxicity of acetaminophen [16]. According to these studies, the lack of MSRA in the *MSRA* KO liver causes elevation of thioredoxin reductase type 1 that is associated with an enhanced sensitivity to the toxic effect of acetaminophen [45]. In addition, MSRB1 deficiency compromises the antioxidant capability of the liver, and thus increases its vulnerability to acetaminophen toxicity. The information about the protective effect of MSRA against kidney damage is currently limited. One study demonstrated that MSRA is able to protect kidneys against cisplatin-induced methionine oxidation and cytotoxicity [46]. Further studies should be conducted to better understand the effect of the mouse strain, the toxic reagent dose, and the mouse age in order to determine the significance of the above toxic compounds to human liver toxicity.

### 3.6. Role of MSRA and MSRB in Cancer

Both MSRA and MSRB have been shown to affect the progression of cancer in various cancerous cell lines. For example, the transcription of *MSRA* is decreased in metastatic hepatocellular carcinoma relative to non-metastatic liver cancer, implying that MSRA may function as a metastasis suppressor [18]. In lungs, the transcription of *MSRA* is decreased in cancerous tissues and it is exacerbated as the function of the breast cancer severity [17]. Lower levels of MSRA transcript in the metastatic MDA-MB231 breast cancer cells causes an increase in cell proliferation and degradation of the extracellular matrix [17]. Additionally, epigenetic regulation may also decrease the transcription of *MSRB1* in the MDA-MB231 cell line [17]. In contrast to the effects of *MSRA* knockdown in cancerous cells, *MSRB1* knockdown in osteosarcoma U2OS cell line decreased cell proliferation and xenograft models and alters the mitogen-activated protein kinase pathway by interfering with the phosphorylation of the key signal transduction proteins [47]. Likewise, *MSRB3* knockdown in several other human cancer cell lines reduces cell proliferation while the overexpression of *MSRB3* stimulates these cell’s proliferation [48]. Furthermore, *MSRB3* knockdown induces cancer cell apoptosis through endoplasmic reticulum stress-dependent pathways [48]. Interestingly, several cancer genomic databases show that the transcription of both the transcription factor *ZEB1* and *MSRB3* are correlated in breast cancer [49]. This phenomenon may be indicative of the tight regulation of MSRB3 transcription by specific transcription factors that are affected by the environment or oxidative stress conditions.

### 3.7. Role of MSRA and MSRB in Hearing Loss

Only recently research has been conducted to determine the role of MSR system in protecting against hearing loss, demonstrated by cochlear damage. In humans, mutations of the *MSRB3* gene are usually linked to autosomal recessive pre-lingual individual deafness [50]. Supportive evidence for the role of MSRB3 in hearing loss is demonstrated by the enhanced apoptosis in the Change reference 52 to 50 part of the ear and deafness in mice, in which their *MSRB3* gene has been disrupted [51]. This research showed the role that *MSRB3* plays in congenital deafness.

Our recent studies show that along with MSRB3, MSRA plays an important role in maintaining the basic cochlea structure, including spiral ganglion neurons and the type IV fibrocytes, which all facilitate auditory signals in mammals. Furthermore, *MSRA* KO mice exhibit significant loss of both of spiral ganglion cells and fibrocystic class IV [52]. These deficiencies can contribute to the sensorineural hearing loss in the high-frequency region. Establishing the role of MSRA as a protector against cochlear hearing loss may provide a better understanding of the involvement of methionine oxidation and its reversal in maintaining adequate hearing capabilities under the conditions of oxidative stress and of acoustic trauma.

## 4. Conclusions

The MSR system mediates the “repair” of oxidized proteins through conversion of MetO to Met residue. This process involves many substrates whose function may be altered due to this MSR activity. Ageing and oxidative stress are triggers for upregulating the cellular antioxidant defense system, including the MSR system. However, when either the upregulation of the MSR system is inadequate or compromised, a disease process may start depending on the cellular/organ condition and the effect of methionine oxidation on protein structure/function. Accordingly, and as described in this review, MSRA/B play a role in various diseases through their basic MetO-reduction activity. It is hoped that novel MSR-based therapies against the described diseases/disorders above; once more knowledge about the MSR system, and in particularly MSRA, will become available. Recently, we have discovered another function of MSRA that is linked to the ubiquitin-like mediated degradation of proteins in *Archaea* [53]. This novel feature of MSRA suggests a dual function for this enzyme that is supposed to determine whether to repair or to degrade an oxidized protein. Recently, we have discovered that MSRA is involved in the ubiquitination of 14-3-3 proteins in the mouse brain [54]. This observation hints at the role of MSRA in protein degradation through the ubiquitin system. It is yet to be determined if this new function of MSRA exists in other eukaryotes and determine the identity of other potential substrates for this MSRA activity. Further evidence for the involvement of MSRA in the ubiquitin–mediated posttranslational modification of proteins will prompt new lines of investigations on the link between MSRA, oxidative stress, and protein ubiquitination in health and disease.
